# Totally implantable venous access port insertion via open Seldinger approach of the internal jugular vein—a retrospective risk stratification of 500 consecutive patients

**DOI:** 10.1007/s00423-021-02097-w

**Published:** 2021-02-07

**Authors:** Felix Becker, Lennart A. Wurche, Martina Darscht, Andreas Pascher, Benjamin Struecker

**Affiliations:** Department of General, Visceral and Transplant Surgery, University Hospital Münster, Waldeyerstrasse 1, 48149 Münster, Germany

**Keywords:** Seldinger, TIVAP, Oncology, Parenteral nutrition

## Abstract

**Purpose:**

Modern oncological treatment algorithms require a central venous device in form of a totally implantable venous access port (TIVAP). While most commonly used techniques are surgical cutdown of the cephalic vein or percutaneous puncture of the subclavian vein, there are a relevant number of patients in which an additional strategy is needed. The aim of the current study is to present a surgical technique for TIVAP implantation via an open Seldinger approach of the internal jugular vein and to characterize risk factors, associated with primary failure as well as short- (< 30 days) and long-term (> 30 days) complications.

**Methods:**

A total of 500 patients were included and followed up for 12 months. Demographic and intraoperative data and short- as well as long-term complications were extracted. Primary endpoint was TIVAP removal due to complication. Logistic regression analysis was used to analyze associated risk factors.

**Results:**

Surgery was primarily successful in all cases, while success was defined as functional (positive aspiration and infusion test) TIVAP which was implanted via open Seldinger approach of the jugular vein at the intended site. TIVAP removal due to complications during the 1st year occurred in 28 cases (5.6%) while a total of 4 (0.8%) intraoperative complications were noted. Rates for short- and long-term complications were 0.8% and 6.6%, respectively.

**Conclusion:**

While the presented technique requires relatively long procedure times, it is a safe and reliable method for TIVAP implantation. Our results might help to further introduce the presented technique as a secondary approach in modern TIVAP surgery.

## Introduction

The incidence of malignant diseases is rising worldwide, with an estimate of over 18 million new annual cancer cases [1]. In parallel, further advances in modern oncological treatment algorithms have led to increasing numbers of cancer patients with an indication for intravenous chemotherapy. As a prerequisite, most patients need a central venous device (CVD). To reduce infectious complications, long-term CVDs are used in form of a totally implantable venous access port (TIVAP), consisting of an easily accessible subcutaneous reservoir port connected to a catheter in a peripheral vein, which tip ends in the vena cava. In addition to administration of intravenous chemotherapy, TIVAPs are frequently used for repeated administration of parenteral nutrition or blood products as well as intravenous medication and periodic blood sampling. Accordingly, an increase in long-term CVD procedures of over 300% was noted in the last two decades, with 808,071 annual procedures in the USA and more than 100.00 procedures in Germany alone [2, 3].

Since the first report of a TIVAP in 1982 by Niederhuber [4], various forms and implantation techniques have been described. In general, the most commonly used access sides to the superior vena cava are the internal jugular and subclavian as well as cephalic vein. In terms of implantation techniques, there are two basic forms of access: on the one hand side, closed/percutaneous procedures via puncture of the subclavian or internal jugular vein and on the other hand side open procedures with either surgical cutdown of the cephalic vein or open Seldinger technique of the internal jugular vein. Among surgeons, the most commonly used location for TIVAP is the cephalic vein and the preferred surgical method is the cutdown approach. This method combines relatively high success rate, easy surgical access, and good long-term function [5].

However, the cephalic vein can be absent or too small as well as technically impossible to use due to an inability to advance a wire or catheter [6]. Overall, a primary failure rate for the cephalic vein cutdown approach is reported to be between 6 and 30% [7, 8]. Therefore, additional rescue strategies have to be in place for TIVAP surgery. Among the reported methods are modified techniques for the cephalic vein or puncture of the subclavian vein on the same side [6]. If both methods fail, a change of surgical side and technique has to be considered. In this case, the open Seldinger approach via the internal jugular vein becomes an option to establish a TIVAP.

However, there is a current gap in our knowledge regarding safety, feasibility, and success of the open Seldinger approach via the internal jugular vein as a form of rescue approach in TIVAP surgery. The aim of the current study is to present a safe and fast surgical technique for TIVAPs via open Seldinger approach of the internal jugular vein and to characterize risk factors associated with primary failure as well as short- (< 30 days) and long-term (> 30 days) complications. Our results might help to further introduce this technique as a secondary approach in modern TIVAP surgery.

## Materials and methods

### Study design

The study design was a retrospective single center study on risk stratification for outcomes after TIVAP via open Seldinger approach of the jugular vein at the department of General, Visceral and Transplant Surgery, University Hospital Münster, Germany. Follow-up period was 12 months. Ethical approval for the study was obtained from the local ethics committee (Ethik-Kommission der Ärztekammer Westfalen-Lippe und Westfälischen Wilhelms-Universität, No. 2018-597-f-S). Prior to analysis, all data were de-identified. The study was conducted in accordance with the ethical principles stated in the Declaration of Helsinki.

### Study population

A total of 500 consecutive cases were included and retrospective patient recruitment ranged from January 2016 to December 2017. Eligible patients were identified via automated electronic chart review based on the respective operative code. Exclusion criteria were age under 18, any intended procedure other than open Seldinger approach via the jugular vein, or an incomplete data set. During the respective study period, TIVAP via open Seldinger approach of the jugular vein was the standard approach in the Department and thus, every TIVAP was scheduled for this approach.

The following data was collected from patients’ charts: demographic data (age, sex, height, weight, and body mass index (BMI)), epidemiologic data (American Society of Anesthesiologists (ASA) physical status classification, alcohol or nicotine use, secondary diseases, and colonization with multidrug-resistant gram-negative bacteria (MDRGN)). MDRGN colonization was assessed by the last nasopharyngeal and/or anal swab before surgery. Swabs are routinely taken from patients when admitted to the hospital or, in case of ambulatory surgery, when patients were evaluated, usually 7 days before surgery. Preoperative laboratory results were reviewed for hemoglobin, leucocyte count, thrombocyte count, international normalized ratio, and partial thromboplastin time. In addition, primary diagnosis and indication for TIVAP (e.g., cancer and chemotherapy or short bowel syndrome and parenteral nutrition) and any previous TIVAP attempts were recorded. For cancer patients, data on pre- or postoperative chemotherapy, radiotherapy, or chemoradiotherapy was extracted. Regarding intraoperative data, success or change of procedure, surgeon’s experience, operation length, intraoperative complications, and type of anesthesia were analyzed.

### Outcome measures

Primary outcome was removal of the TIVAP due to complications (grade IIIa and IIIb according to Clavien-Dindo) during the 12 months follow-up time. Secondary outcomes included unsuccessful TIVAP implantation via open Seldinger approach of the jugular vein and switch to an alternative approach as well as any postoperative complication (grades II–V according to Clavien-Dindo). Complications were divided into short-term (first 30 postoperative day) and long-term complications (days 31 to 365 after surgery). Complications included were hemorrhage, infection, thrombosis, dislocation, dysfunction, and any other causes of admission to our center related to TIVAP.

### Surgical technique

**Fig. 1:**
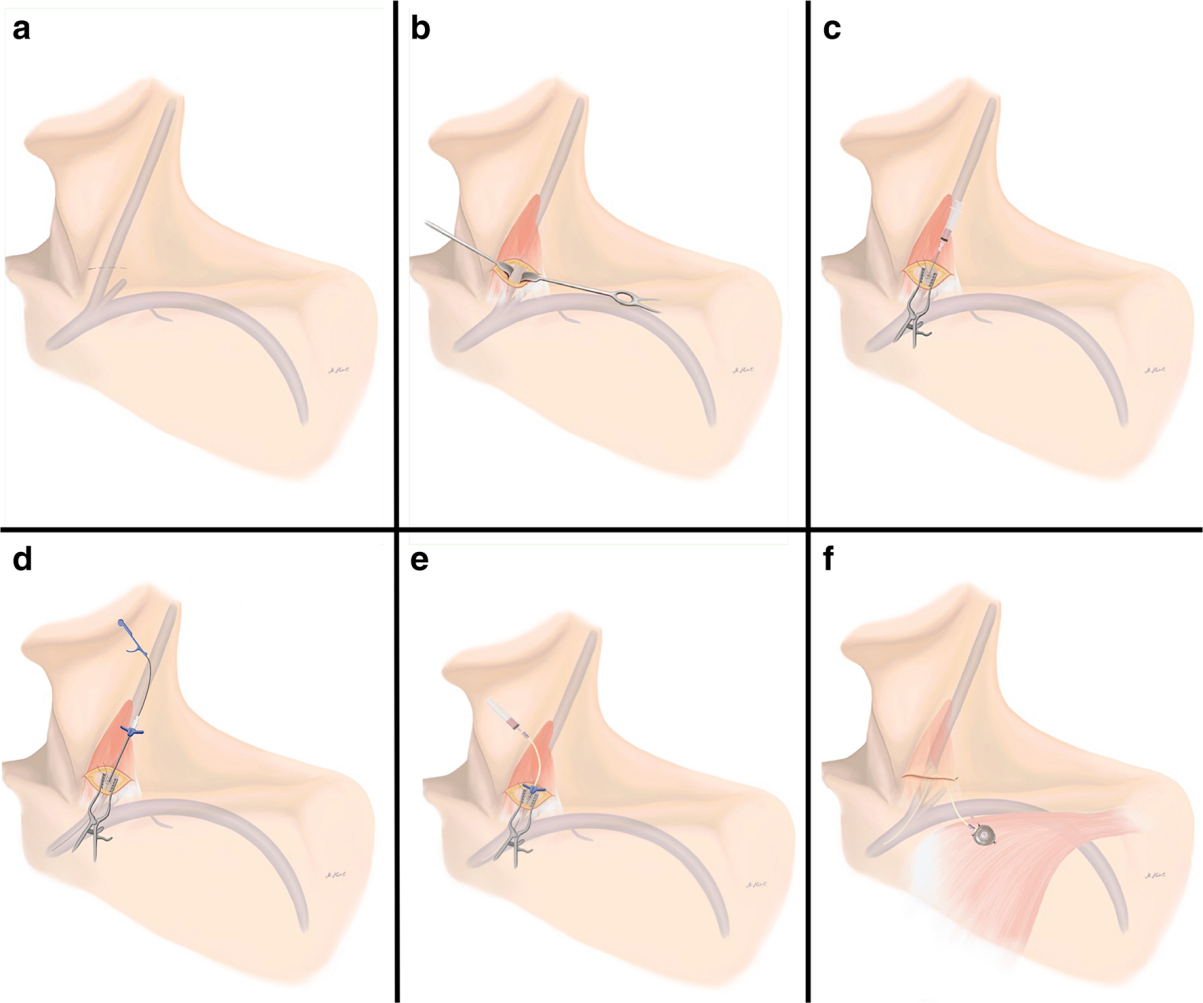
Schematic overview of TIVAP insertion via open Seldinger technique of the internal jugular vein. **a** A 2.5-cm-long horizontal incision, 2 cm above and parallel to the clavicle right above the sternocleidoide muscle, is used. **b** The medial and lateral body of the sternocleidomastoid muscle are divided with hooks at the fossa supraclavicularis minor, where the internal jugular vein is exposed. **c** A purse string suture is conducted with a resorbable suture and the vein is punctured. **d** After successful puncture, a guidewire and subsequently a vascular dilator with a peel-away sheath are introduced. **e** Following removal of the dilator and wire, the catheter is introduced through the peel-away sheath, which is then retracted. **f** Blunt dissection is used to create a pouch for the port chamber 3 cm below the clavicle and the chamber is fixed at the pectoral fascia

Standard preoperative work-up for every patient scheduled for TIVAP insertion via open Seldinger technique of the internal jugular vein included ultrasound of the cervical vessels and a blood test including at least a blood count, international normalized ratio, and partial thromboplastin time. Depending on the patient’s condition, implantation was conducted under either general or regional anesthesia. Preferred site of implantation was the right side. The standard operation included a 2.5-cm-long horizontal incision, about 2 cm above and parallel to the clavicle right above the sternocleidoide muscle (Fig. 1). Then, the subcutaneous tissue and the platysma were split, and the medial and lateral body of the sternocleidomastoid muscle were divided with hooks at the fossa supraclavicularis minor, where the internal jugular vein was exposed after preparation. Then, a purse string suture was completed with a resorbable suture and the vein was punctured following TIVAP-catheter insertion in typical Seldinger technique. Correct catheter position was determined with a mobile X-ray device. Next, blunt dissection was used to create a pouch for the port chamber 3 cm below the clavicle and the chamber was fixed at the pectoral fascia. After wound closure, TIVAP was standardized tested with blood aspiration and flushing via transcutaneous puncture.

### Statistical analysis

Statistical analysis was conducted with IBM SPSS Statistics 25 (IBM, Armonk, NY, USA). Biometrical and epidemiological data as well as surgical data and complications were analyzed using univariate analysis. For the primary endpoint, removal of the TIVAP due to complications during the 12 months follow-up time, the confidence interval for the probability was calculated using the Clopper-Pearson interval. To correlate the primary endpoint with possible risk factors, a logistic regression analysis was used. Due to the given case number and incidence of complications, five independent variables were deemed appropriate for logistic regression analysis, based on clinical experience and potential usefulness for future clinical decision-making. The independent variables chosen were colonization with MDRGN bacteria, BMI, white blood cell (WBC) count, international normalized ratio, and diabetes mellitus. Leucocyte count, INR, and BMI were defined as metric while MDRGN and diabetes mellitus were defined as nominal variables. In the logistic, MDRGN and diabetes mellitus were included as categorical and INR, BMI, and leucocyte count were included as continuous. To further dissect the influence of the surgeon’s experience, a second iteration of the logistic regression model was conducted, using only the surgeon’s experience as independent variable. Surgeon’s experience was defined as an ordinal variable and included as categorical in the logistic regression analysis.

## Results

### Study population characteristics

Five hundred patients between January 2016 and December 2017 were found eligible and included in the analysis. The majority of patients were female (52.8%), average age at surgery was 56.5 ± 15.1 years (ranging from 18 to 87 years) and mean BMI was 25.4 ± 5.7 (ranging from 14.4 and 58.1). Patient’s ASA classification ranged from 1 to 4 with 96.4% of all patients having a classification of 2 or 3 (ASA 1: 3.0%; ASA 2: 59.4%; ASA 3: 37.0%; ASA 4: 0.6%). Leading primary diagnosis was malignancy in 96.6% (65% solid, 31.6% non-solid), followed by autoimmune and rare diseases (2.8%) and malabsorption in three cases (0.6%). Thus, main indication for TIVAP was intravenous application of chemotherapy (95.4%), followed by parenteral nutrition (2.4%) and other intravenous medications including immunotherapy (2.0%) as well as photopheresis (1.4%). Four patients received chemotherapy and parenteral nutrition while two patients received parenteral nutrition and other medications. The prevalence of secondary diagnoses in the patient group was 11.2% for coronary heart disease, 3.0% for chronic obstructive pulmonary disease, 7.2% for diabetes mellitus, 28.8% for arterial hypertension, and 5.4% for chronic kidney disease (Table 1).

**Table 1:** Patient characteristics of 500 TIVAP recipients

Patient characteristics	
Age (mean ± SD)	56.5 ± 15.1
Gender (% males)	47.2
BMI (mean ± SD	25.4 ± 5.7
ASA score (%)	
1	3.0
2	59.4
3	37.0
4	0.6
Primary diagnosis (%)	
Solid malignant cancer	65.0
Non-solid malignant cancer	31.6
Autoimmune and rare diseases	2.8
Malabsorption	0.6
Secondary diagnosis (%)	
aHT	28.8
CHD	11.2
Type 2 DM	7.2
CKD	5.4
COPD	3.0

Results are presented as mean ± standard deviation (SD) or relative frequencies. *ASA* American Society of Anesthesiologists physical status, *aHT* arterial hypertension, *CHD* coronary heart disease, *DM* diabetes mellitus, *CKD* chronic kidney disease, *COPD* chronic obstructive pulmonary disease

Surgery was primarily successful in all cases, while success was defined as a functional (positive aspiration and infusion test) TIVAP which was implanted via open Seldinger approach of the jugular vein at the intended site. Of the 500 procedures, 10.8% were done in local anesthesia and 17.2% in local anesthesia and sedation while 72.0% were conducted under general anesthesia. Duration of surgery ranged from 14 to 175 min with a mean of 35.9 ± 15.8 min. Intraoperative complications occurred in four cases: one lesion of the carotid artery due to accidental puncture with the needle which needed suture, one bleeding of the tissue while exposing the jugular vein which needed extended hemostasis and suture, one intubation of the patient due to agitation and hyperventilation during surgery in local anesthesia, and one pneumothorax due to pleural laceration and subsequent chest-drain (Table 2).

**Table 2:** Perioperative characteristics of 500 TIVAP procedures

Operative characteristics	
Primary success rate (%)	100
Side (% right)	74.8
Length of procedure (min, mean ± SD)	35.9 ± 15.8
Intraoperative complications (*n*, %)	4 (0.8)
Type of anesthesia (%)	
Local anesthesia	10.8
Local anesthesia and sedation	17.2
General anesthesia	72.0
Surgeon board certified (%)	
Yes	26.8
No	73.2

Results are presented as mean ± standard deviation (SD) or relative frequencies

When analyzing the surgeon’s experience, it was found that 134 procedures were done by an assistant physician (26.8%), 263 by a specialist physician (52.6%), and 103 by a senior or chief physician (20.6%) (Table 2). Conducting a second logistic regression model to further investigate the influence of the surgeon’s experience, it was found that the surgeon’s experience as an independent factor showed no significant influence (*p* > 0.05) and the experience of the surgeon had no effect on the probability of removal with an odds ratio of − 0.033. The logistic regression model with the surgeon’s experience as an independent factor showed no significant influence (*p* > 0.05) and the experience of the surgeon had no effect on the probability of removal with an odds ratio of − 0.033. Univariate analysis of the complication rate of each physician group showed the following differences: TIVAPs done by assistant physicians having a removal rate of 5.2%, TIVAPs done by specialist physicians a rate of 5.3%, and the TIAPs done by senior or chief physicians a rate of 4.9%

In the 30-day follow-up after surgery, there were four complications with one TIVAP removal. In three cases, wound infection occurred, of which two could be successfully treated with antibiotics while one resulted in TIVAP removal. In one case, hemorrhage occurred at the day of surgery, which needed revision surgery for hemostasis while the TIVAP could be preserved (Table 3).

**Table 3:** Short- as well as long-term complications and TIVAP removal rates

Postoperative complications and removal rates			
	30 days (*n*)	1 year (*n*)	Catheters removed (*n*, %)
Infection	3	22	23 (4.6)
Hemorrhage	1	2	1 (0.2)
Dysfunction	0	5	3 (0.6)
Thrombosis	0	2	0 (0)
Dislocation	0	2	1 (0.2)

Results are presented as relative frequencies

After the first 30 postoperative days and until the end of follow-up after 12 months, 33 patients were referred to our center with TIVAP complications. In 22 cases, an infection had occurred, which resulted in 22 TIVAP removals. In two patients, late TIVAP-associated hemorrhage occurred and one patient was scheduled for removal. In addition, TIVAP dysfunction in five cases was recorded, which led to removal in three patients. Two cases of thrombosis were diagnosed and could be successfully treated with lysis while dislocation occurred in two patients of which one needed TIVAP removal (Table 3). No case of pinch-off syndrome occurred.

Accumulated, this leads to a TIVAP removal rate due to complication during the 1st year follow-up of 5.6% (28 cases). The estimated probability of removal due to complication therefore was between 3.4 and 7.5% using the Clopper-Pearson interval with a 0.95 confidence level (Table 4).

**Table 4:** Probability of TIVAP removal

Probability of TIVAP removal			
Follow-up	Complication rate (%)	Removal rate (%)	Clopper-Pearson interval (0.95 CI)
30 days	0.8	0.2	0.0–1.1%
1 year	6.6	5.4	3.3–7.3%
Accumulated	7.4	5.6	3.4–7.5%

Results are presented as relative frequencies. *CI* 95% confidence interval

When analyzing the profile of the investigated independent risk factors, it was found that mean WBC ranged from 0.23 to 101.00 (× 10^9/L) while mean INR was 1.0 ± 0.18 (ranging from 0.81 to 3.62). A total of 36 (7.2%) patient had diabetes mellitus, 13.6% were colonialized with MDRGN bacteria and mean BMI was 25.4 ± 5.7 kg/m^2^. In the logistic regression model, none of the five dependent variables had a significant influence on the probability of a TIVAP removal. Nevertheless, three variables had nearly no influence: INR, BMI, and WBC with an odds ratio of − 0.030, 0.025, and 0.019, respectively. Colonization with MDRGN bacteria had a negative (meaning theoretically protective) influence with an odds ratio of − 0.488. Diabetes had a positive (meaning risk factor) influence on the probability of removal with an odds ratio of 1.285 (Table 5).

**Table 5:** Logistic regression model for risk factors of TIVAP removal

Risk factors of TIVAP removal		
Independent factor	OR (0.95 CI)	*p* value
BMI	0.025 (0.960–1.094)	0.456
WBC count	0.019 (0.988–1.050)	0.230
INR	− 0.030 (0.088–10.643)	0.980
Type 2 DM	1.285 (0.290–18.007)	0.433
MDRGN colonization	− 0.669 (0.195–1.346)	0.175

*BMI* body mass index, *WBC* white blood cell, *INR* international normalized ratio, *DM* diabetes mellitus, *MDRGN* multidrug-resistant gram-negative bacteria, *OR* odds ratios, *CI* 95% confidence interval

## Discussion

Modern oncological treatment strategies demand a safe and reliable permanent venous access rout. Thus, TIVAP implantation is among the most commonly performed procedures in the western world. Accordingly, numerous publications including randomized controlled trials have been conducted to identify a favorable access side and superior implantation method. In a recent Cochrane analyses, Hsu et al. reviewed nine randomized controlled trials encompassing a total of 1253 patients to identify the optimal evidence-based method for TIVAP implantation [9]. Yet, the authors had to conclude that the current available evidence is insufficient to draw a definitive conclusion. This may explain existing regional differences, with the cutdown method (performed by surgeons) being the favorite method in Germany [10], while globally the percutaneous approach (performed by interventional radiologists, surgeons, anesthesiologists) is considered to be the most common technique for TIVAP implantation [11, 12].

From a surgical standpoint, the cutdown method of the cephalic vein is a safe, easy, and standardized operation with excellent short- and long-term results. However, there are numerous scenarios in which an additional TIVAP strategy is needed. The cephalic vein can be absent or too small or even closed during previous TIVAP implantation. If the surgical option of cephalic vein cutdown becomes absent, one has to carefully consider between an alternative open approach and a percutaneous technique. Di Carlo et al. reviewed 11,381 TIVAP patients (6535 percutaneous procedures, 4846 open procedures) over 27 years and found an increase in early complications in patients with a percutaneous technique compared with surgical cutdown (4.5% vs. 0.9%, respectively), including pneumothorax, hemothorax, and arterial puncture [12]. Especially in patients with a significant risk for pulmonary decompensation, percutaneous puncture of the subclavian vein with the risk for pneumothorax (up to 4.3%) [13] can be considered as too dangerous. In addition, in randomized controlled trials, percutaneous puncture of the internal jugular vein and TIVAP placement via Seldinger technique were reported to have a success rate of only 74% and were accompanied by a complication rate (early and late) of 23.5% [14]. However, there are also undoubted advantages of a percutaneous procedure, particularly since it can be performed under local anesthesia, which reduces the total procedure time (with equivalent operation times), leading to reduced costs. In addition, its safety can be significantly increased when using fluoroscopy and ultrasound, especially when the internal jugular vein is used [15, 16]. Therefore, the percutaneous procedure (using either the internal jugular or subclavian vein) is commonly used as a reliable exit strategy in scenarios where the cutdown technique of the cephalic vein fails or as primary approach, especially by non-surgical health care professionals.

While there are numerous reports regarding the percutaneous Seldinger technique for TIVAP implantation in the internal jugular vein, data on the open Seldinger approach of the internal jugular vein is scare. The current report closes this gap in our knowledge by offering first evidence regarding its technical details, successes rate, safety, and risk factors. To the best of our knowledge, this is the first study providing an in-depth description of this approach as a rescue strategy in modern TIVAP surgery.

First and foremost, the data presented in this study allows for the conclusion that TIVAP insertion via an open Seldinger approach of the internal jugular vein is a safe and reliable method and provides value as an exit strategy. In line with this, the use of the Seldinger technique instead of direct catheter insertion provides an additional tool to increase the safety since the correct position of a guidewire in the superior vena cava can be controlled by intraoperative fluoroscopy. Therefore, this technique is safe and reliable, especially in patients with a complex anatomy. It is notable that, with the here described technique, a success rate of 100% was achieved and that short- and long-term complications (0.8 and 6.6%, respectively) as well as intraoperative complication rates (0.8%) were comparable to previous reports [17]. This is especially important in oncological patients, in which any TIVAP-related complication or failure of implantation can postpone the start or continuity of intravenous chemotherapy. Therefore, TIVAP removal due to complications during the 12 months follow-up time was chosen as the primary endpoint and an accumulated removal rate of 5.6% (with an estimated probability of removal due to complication between 3.4 and 7.5% using the Clopper-Pearson interval with a 0.95 confidence level) was revealed. In a recent randomized controlled trial, Biffi et al. reported a removal rate of 4.6% for TIVAPs implanted via surgical cutdown of the cephalic vein [17]. Moreover, the here reported removal rate of 5.6% is similar to the results obtained in previous studies. Kock et al. reported a removal rate of 11.9% in a mixed TIVAP cohort (implantation sides included cephalic, subclavian, internal, and external jugular as well as great saphenous vein) while Nagasawa et al. described a removal rate of 8% and 8.2% for TIVAP implantation in the internal jugular or subclavian vein, respectively [15, 18]. In comparison, Ahn et al. revealed a removal rate due to complications of 3% in a cohort of 1254 TIVAPs, all implanted in the internal jugular vein in an ultrasound guide percutaneous technique [19].

In addition, although surgically somewhat more challenging than the venous cutdown technique, our data demonstrates that this procedure can be conducted by junior surgeons (guided by an experienced colleague) since the surgeon’s experience seems to have only a limited effect on the probability of removal. When regarded as an exit strategy in TIVAP surgery, it is reasonable to assume that this technique is employed in rather high-risk patients. Therefore, the conducted risk analysis is reassuring by demonstrating that the distribution of established risk factors (MDRGN bacteria, BMI, WBC count, international normalized ratio, and diabetes mellitus) among TIVAP patients had no significant influence on the probability of a TIVAP removal.

One of the disadvantages of the described procedure is the relatively long procedure time (35.9 ± 15.8 min), compared to average implantation time of 21 min for the venous cutdown method in a similar cohort [5]. An additional disadvantage of the here presented technique is the high rate of procedures performed under general anesthesia (72%), compared to the other TIVAP procedures, which can be predominantly performed under regional anesthesia. Thus, the here described technique requires a longer total procedure time with more complex logistics, and thus higher costs. In a comparable cohort, Hüttner et al. found that in 1159 patients (583 patients with surgical cutdown of the cephalic vein and 576 patients with percutaneous puncture of the subclavian vein guided) only 6.1% underwent TIVAP under general anesthesia [20]. In addition, it was not analyzed whether TIVAP implantation was routinely scheduled or conduced as an urgent procedure to allow for rapid start of chemotherapy in critical cases. Although this might be a minor bias, time of operation has been proven to affect outcomes of surgical procedures [21–23].

## Conclusion

To summarize, this study provides evidence from retrospectively analyzing records of a large patient cohort who received a TIVAP via an open Seldinger approach of the internal jugular vein. The results presented here elucidate three main findings. First, the described procedure is safe and reliable, a statement which is based on a 100% success rate and rare intraoperative complications. Second, for patients with an eminent need for a reliable venous access, the here presented technique is applicable since it combines low short- as well as long-term complications with a high intraoperative success rate. Third, when conducted in the here described perioperative work-up, the 1-year durability is independent from risk factors such as BMI or MDRGN bacteria colonization. In conclusion, the open Seldinger approach of the internal jugular vein for TIVAP implantation is a reasonable second-line strategy for patients in which the surgical cutdown of the cephalic vein is impossible and should be part of every surgeon’s skill reservoir.

## Data Availability

All relevant data is within the manuscript.

## Declarations

### Disclaimer

The funders had no role in study design, data collection and analysis, decision to publish, or preparation of the manuscript.
